# SINGLE-CELL TRANSCRIPTOMICS OF AGING MOUSE ISLET REVEALS AGE-RELATED RECRUITMENT OF ISLET-RESIDENT MACROPHAGE

**DOI:** 10.1093/geroni/igz038.960

**Published:** 2019-11-08

**Authors:** Jia Nie, Nicolas Musi

**Affiliations:** 1 University of Texas Health Science Center Barshop Aging Institute, San Antonio, Texas, United States; 2 Barshop Institute for Longevity and Aging Studies, University of Texas Health Science Center, San Antonio, Texas, United States

## Abstract

Type 2 diabetes (T2D) prevalence increases with age. The notion of inevitable progression of T2D has been challenged by reports of remission in some human T2D cases; however, this remission is dependent on islet function reserve. To elucidate the molecular mechanisms driving islet cell dysfunction, it is necessary to understand islet cell composition, diversity, and function throughout the lifespan. We generated a single-cell transcriptomic atlas of healthy islets isolated from young (5 weeks old), middle-aged (12 months old), and older-aged (25 months old) mice. Cell clustering identified 13 initial cell clusters that were further sub-clustered. This single-cell RNAseq profile showed that each cell type/group has different markers and functional characteristics and that age causes a remarkable shift in islet cell composition, diversity, and number. By comparing macrophages from young and old mice, we also found that aged islets contain a higher number of islet-resident macrophages. Overall, this single-cell islet atlas covers nearly all cells in the normal islet and allows a comprehensive exploration of all transcriptional states throughout the lifespan.

